# Role of 3'-Deoxy-3'-[^18^F] Fluorothymidine Positron Emission Tomography-Computed Tomography as a Predictive Biomarker in Argininosuccinate Synthetase 1-Deficient Thoracic Cancers Treated With Pegargiminase

**DOI:** 10.1016/j.jtocrr.2022.100382

**Published:** 2022-07-20

**Authors:** Teresa A. Szyszko, Joel T. Dunn, Melissa M. Phillips, John Bomalaski, Michael T. Sheaff, Steve Ellis, Lucy Pike, Vicky Goh, Gary J.R. Cook, Peter W. Szlosarek

**Affiliations:** a King’s College London and Guy’s and St Thomas’ PET Centre, St Thomas’ Hospital, London, United Kingdom; b Department of Nuclear Medicine, Royal Free Hospital NHS Trust, London, United Kingdom; c Department of Oncology, University College London, London, United Kingdom; d Department of Medical Oncology, St. Bartholomew’s Hospital, Barts Health NHS Trust, London, United Kingdom; e Polaris Pharmaceuticals Inc., San Diego, California; f Department of Histopathology, Royal London Hospital, Barts Health NHS Trust, London, United Kingdom; g Department of Diagnostic Imaging, St. Bartholomew's Hospital, Barts Health NHS Trust, London, United Kingdom; h Cancer Imaging, School of Biomedical Engineering and Imaging Sciences, King’s College London, London, United Kingdom; i Centre for Cancer Biomarkers and Biotherapeutics, Barts Cancer Institute, Queen Mary University of London, London, United Kingdom

**Keywords:** FLT PET-CT, Pegargiminase, Arginine, ASS1, Thoracic cancers

## Abstract

**Introduction:**

Pegargiminase (ADI-PEG 20I) degrades arginine in patients with argininosuccinate synthetase 1-deficient malignant pleural mesothelioma (MPM) and NSCLC. Imaging with proliferation biomarker 3'-deoxy-3'-[^18^F] fluorothymidine (^18^F-FLT) positron emission tomography (PET)-computed tomography (CT) was performed in a phase 1 study of pegargiminase with pemetrexed and cisplatin (ADIPemCis). The aim was to determine whether FLT PET-CT predicts treatment response earlier than CT.

**Methods:**

A total of 18 patients with thoracic malignancies (10 MPM; eight NSCLC) underwent imaging. FLT PET-CT was performed at baseline (PET1), 24 hours post-pegargiminase monotherapy (PET2), post one cycle of ADIPemCis (PET3), and at end of treatment (EOT, PET4). CT was performed at baseline (CT1) and EOT (CT4). CT4 (modified) Response Evaluation Criteria in Solid Tumors (RECIST) response was compared with treatment response on PET (changes in maximum standardized uptake value [SUVmax] on European Organisation for Research and Treatment of Cancer–based criteria). Categorical responses (progression, partial response, and stable disease) for PET2, PET3, and PET4 were compared against CT using Cohen’s kappa.

**Results:**

ADIPemCis treatment response resulted in 22% mean decrease in size between CT1 and CT4 and 37% mean decrease in SUVmax between PET1 and PET4. PET2 agreed with CT4 response in 62% (8 of 13) of patients (*p* = 0.043), although decrease in proliferation (SUVmax) did not precede decrease in size (RECIST). Partial responses on FLT PET-CT were detected in 20% (3 of 15) of participants at PET2 and 69% (9 of 13) at PET4 with good agreement between modalities in MPM at EOT.

**Conclusions:**

Early FLT imaging (PET2) agrees with EOT CT results in nearly two-thirds of patients. Both early and late FLT PET-CT provide evidence of response to ADIPemCis therapy in MPM and NSCLC. We provide first-in-human FLT PET-CT data in MPM, indicating it is comparable with modified RECIST.

## Introduction

Arginine is critical for the growth of many human cancers.^1^ It is involved in multiple aspects of tumor metabolism, including synthesis of proteins, nucleotides, nitric oxide, polyamines, proline, and glutamate. Loss of the tumor suppressor argininosuccinate synthetase 1 (ASS1) results in arginine auxotrophy, typical of chemoresistant, poor prognosis cancers, including hepatocellular carcinoma, melanoma, thoracic and urological cancers, and sarcomas.^2^, ^3^, ^4^, ^5^, ^6^, ^7^ Mechanistically, ASS1 loss promotes diversion of the arginine precursor, aspartate, for enhanced pyrimidine synthesis and tumor cell proliferation.^8^ The arginine dependency of ASS1-dysregulated cancers has driven the clinical development of arginine-depleting enzymes as novel antimetabolites, including pegargiminase or pegylated arginine deiminase (ADI-PEG 20, ADI).^9^

Several groups have used molecular imaging for response assessment to arginine-deprivation therapy. Early metabolic responses with F-18 fluoro-2-deoxy-D-glucose (FDG) positron emission tomography (PET)-computed tomography (CT) to ADI-PEG 20 monotherapy were evaluated in the ADAM trial for malignant pleural mesothelioma (MPM). Although no modified Response Evaluation Criteria in Solid Tumors (mRECIST) partial or complete responses were observed during the study, FDG PET-CT revealed a partial metabolic response in 46%, stable disease (StD) in 31%, a mixed response in 8%, and progressive disease in 15% of patients.^10^ Nevertheless, flare reactions and stunning effects have been described, limiting FDG-PET use as an early metric of treatment response in some circumstances.^11^ Moreover, an increase in FDG uptake post-ADI-PEG 20 therapy has been revealed using a melanoma xenograft mouse model highlighting cell-of-origin as a driver of arginine-based therapeutics.^12^

The TRAP clinical study (Phase 1 Study in Subjects With Tumors Requiring Arginine to Assess ADI-PEG 20 With Pemetrexed and Cisplatin; ClinicalTrials.gov number NCT02029690, registered December 16, 2013) investigated arginine deprivation combination chemotherapy (pegargiminase with pemetrexed and cisplatin [ADIPemCis]) in patients with ASS1-deficient MPM and nonsquamous NSCLC.^13^^,^^14^ This imaging substudy of the dose-expansion TRAP study aimed to assess tumor proliferation as a marker of treatment response in MPM and NSCLC to ADIPemCis. We tested the hypothesis that FLT PET-CT has utility as an early biomarker of response to pegargiminase-based therapy, bypassing the potential limitations of FDG PET-CT of inflammation and that decreased proliferation is predictive of drug efficacy and would precede any change in tumor size.

## Materials and Methods

### Participants

Enrolment of participants into the FLT PET-CT TRAP substudy occurred in a 24-month period from May 2015 to May 2017. A total of 10 participants with histologically proven advanced MPM were included (mean age 69 ± 7.6 y): five with biphasic MPM, four with sarcomatoid MPM, and one with epithelioid MPM. The eight participants with nonsquamous NSCLC all had stage IV disease: seven with adenocarcinoma and one with a giant-cell variant (mean age 58 ± 8.4 y). In addition, three of eight had previous surgery and five of eight had external beam radiotherapy.^15^

Participants were aged 18 years or more, chemotherapy naive with histologically proven ASS1-deficient malignancy (required >50% ASS1 loss to enter the trial). Additional eligibility included an Eastern Cooperative Oncology Group performance status of 0 or 1, no major comorbidities, a minimum expected survival of 3 months, and measurable disease by mRECIST criteria for MPM^16^ and RECIST 1.1 for NSCLC.^17^ ASS1% calculation is described by Beddowes et al.^13^

Exclusion criteria included recent major surgery, history of another active primary cancer, seizures, and previous therapy with pegargiminase. The clinical protocol was approved by Leeds East Research Ethics Committee (14/YH/0090) and was sponsored by Polaris Pharmaceuticals, Inc. Approvals were obtained from the Medicines and Healthcare Products Regulatory Agency and Administration of Radioactive Substances Advisory Committee before study initiation. Informed consent was obtained from each participant. Informed consent was obtained from all individual participants included in the study, and all patients signed written informed consent.

Participants received the maximum tolerated dose derived from the dose-escalation study: weekly intramuscular ADI-PEG 20 (36 mg/m^2^), starting on day 1 with standard doses of pemetrexed (500 mg/m^2^) and cisplatin (75 mg/m^2^) both given intravenously every 21 days, except for cycle 1 where the chemotherapy was administered on day 3 (i.e., 48 h after the ADI-PEG 20).^13^ Participants with MPM received up to a maximum of six cycles of treatment every 3 weeks (up to 18 wk). Participants with NSCLC received up to a maximum of four cycles of treatment (up to 12 wk).

### CT Imaging

Contrast-enhanced (CE) CT imaging was performed as part of routine clinical care: at baseline (CT1), after 2 cycles of treatment (at approximately 6 wk, CT2), and end of treatment (EOT) (at 12 wk for NSCLC and 18 wk for MPM and CT4). In MPM, subjects had an additional clinical CT scan after 4 cycles (at 12 wk, CT3).

#### CT Image Acquisition

Diagnostic CE CTs were acquired as standard of care on a Definition AS 64 slice CT scanner (Siemens Healthcare, Erlangen, Germany). IV CE CT scans of the chest, abdomen, and pelvis (120 kV, 120 mAs) obtained in one continuous volume reconstructed with a slice thickness of 1.5 mm were available for review. Each participant received 80 to 100 mL of IV iodinated contrast medium (iodixanol 300 or iohexol 300) injected at a rate of 2 to 3 mL/s, and scanning began after a delay of 60 seconds.

### PET Imaging

**Figure 1:**
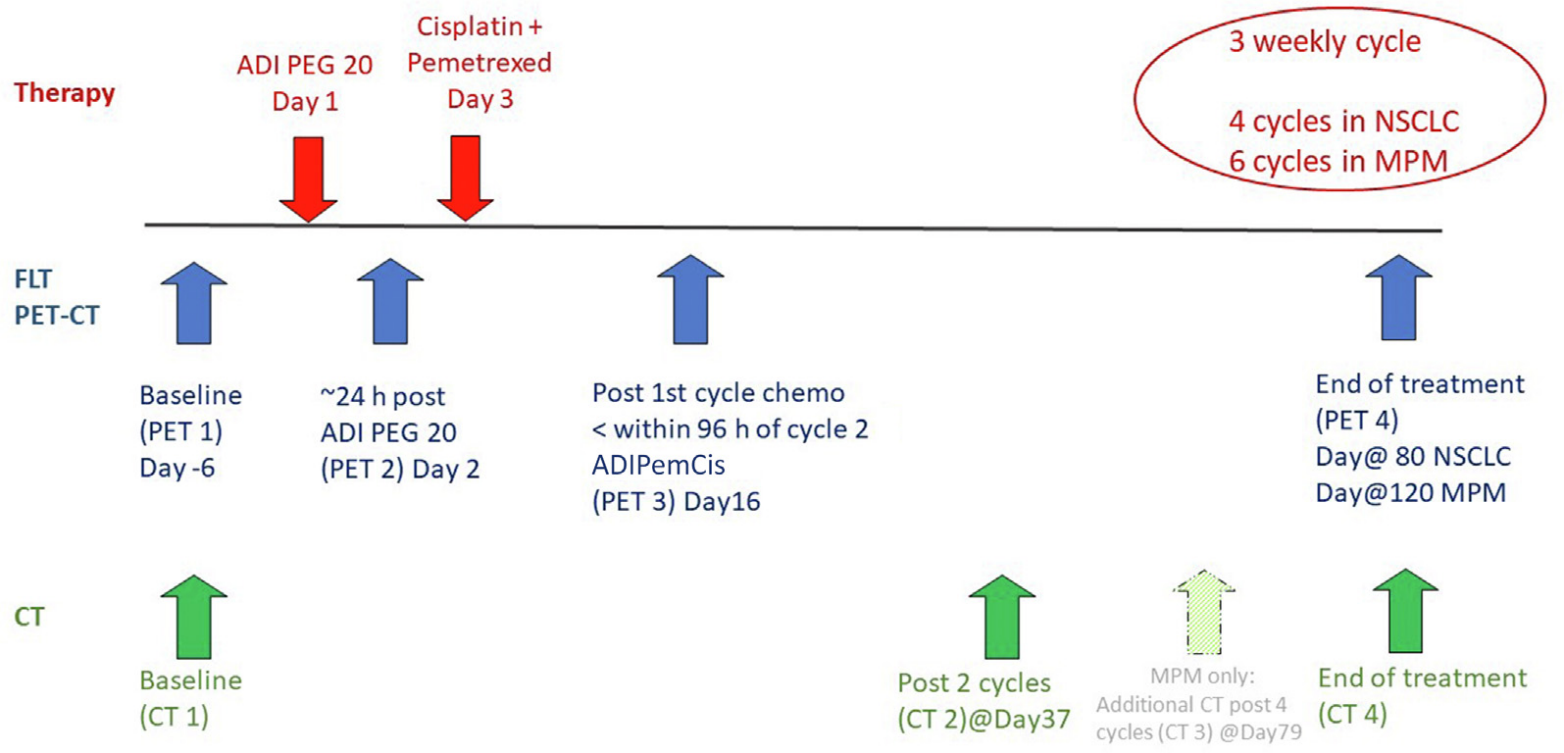
Timing of PET-CT and CT imaging and therapy. The postcycle 4 CT was only done for the MPM participants, and there is a slight difference in the timing of PET 4 in the two groups (at day 120 for MPM and at day 80 for NSCLC). ADI-PEG 20, pegargiminase; ADIPemCis, pegargiminase with pemetrexed and cisplatin; CT, computed tomography; FLT, 3'-deoxy-3'-fluorothymidine; MPM, malignant pleural mesothelioma; PET, positron emission tomography.

FLT PET-CT (with low-dose CT) imaging took place in a longitudinal study with scans as follows: at baseline (PET1); 24 hours after the first dose ADI-PEG 20 but before cisplatin and pemetrexed, day 2 (PET2); after cycle one of ADIPemCis, day 16 (PET3); and at the EOT (PET4), approximately day 120 for MPM and day 80 for NSCLC (Fig. 1).

There was slight variation in the timing of PET2 between groups owing to a change in FLT tracer availability and scheduling: for the NSCLC group, this was at 28 to 29 hours post-ADI; whereas in the MPM group, this was 22 to 24 hours.

#### PET-CT Image Acquisition

Mean administered activity of 244 plus or minus 6.3 MBq of FLT was injected IV in participants who were well hydrated. The PET emission acquisition was started 60 plus or minus 5 minutes after the FLT administration. All PET-CT images were acquired on a GE Discovery 710 PET-CT scanner (GE Healthcare, Chicago, IL). Patients were positioned in the scanner with their arms raised; each scan covered the skull base to the bottom of the liver with an axial field of view of 14.9 cm in four to five bed positions. All PET data were acquired in three-dimensional time-of-flight acquisitions with scan length of 4 minutes per bed position. A low-dose CT scan (140 kV, 10 mA, 0.5 s rotation time, 40 mm collimation) was performed at the start to provide attenuation correction. The PET data were corrected for dead time, scatter, randoms, and attenuation using standard algorithms provided by the scanner manufacturer. Images were reconstructed using iterative reconstruction with time-of-flight (reconstruction parameters: 2 iterations, 24 subsets, Gaussian postfilter with 6.4 mm full width at half maximum, 4 mm voxels). Response scans were all performed at the same time plus or minus 5 minutes after injection as the baseline scan.

### Imaging Analysis

CE CT response was assessed using RECIST 1.1 (NSCLC) and mRECIST (MPM) criteria. Change from CT1 to CT4 was used to define treatment response by RECIST. These are defined as partial response (PR): at least 30% decrease in sum of diameters of target lesion(s); progressive disease (PD): at least a 20% increase in the sum of diameters of target lesion(s); StD: in between. The reference was the baseline study. There was only one tumor assessed for each patient, and there were no cases revealing a complete response. The reference in all cases was the baseline study (CT1).

There are no guidelines for FLT PET-CT response measurement, so an adaption of the European Organisation for Research and Treatment of Cancer (EORTC) criteria developed for FDG^18^ was used as described in the ADAM trial for ADI-PEG 20 monotherapy in MPM.^10^ Volumes of interest were drawn manually using Hermes Gold 3 (Sweden) software within the primary tumor at baseline at the site of most intense uptake (by an experienced radiologist) and then redrawn at the same anatomical location on subsequent scans to measure maximum standardized uptake value (SUVmax). We calculated percentage change in SUVmax from PET1 to time points PET2, PET3, and PET4. PR was defined as 15% decrease in SUVmax, PD 25% increase in SUVmax, and StD in between. Responses on PET were compared with RECIST response. In addition, we looked at treatment response in terms of PR, StD, and PD on PET2, PET3, and PET4 and compared with response to treatment on CT4.

### Statistical Analysis

Statistical analysis was performed using SPSS (IBM SPSS Statistics for Windows, Version 26.0, IBM Corp., Armonk, NY). Concordance of CE CT response from PET scans was assessed by comparing quantitative responses between PET2 to PET4 (percentage change in SUVmax) and CE CT (percentage change in RECIST length) using the nonparametric correlation parameter, Kendall’s tau. Categorical responses (PD, PR, and StD) for PET2, PET3, and PET4 were compared against those from CE CT using Cohen’s kappa. Correlation of survival: overall survival (OS, time from baseline PET to death) and progression-free survival (PFS, time from baseline PET to progression on imaging) were performed using nonparametric (Kendall’s tau) tests. Cox regression was used to investigate association between individual quantitative responses and hazard ratio. A significance threshold of *p* value less than 0.05 was used in all cases.

## Results

### FLT PET-CT SUV Data and Comparison With CE CT

The MPM group mean age was 69 years (range: 58–82 y), 9 of 10 participants were male, and ASS1% loss was 51% to 100% (mean = 79%). The NSCLC group mean age was 58 years (range: 39–65 y), four of eight participants were male, and ASS1% loss was 55% to 100% (mean = 82%). Demographic data are in Supplementary Table 1.

**Figure 2:**
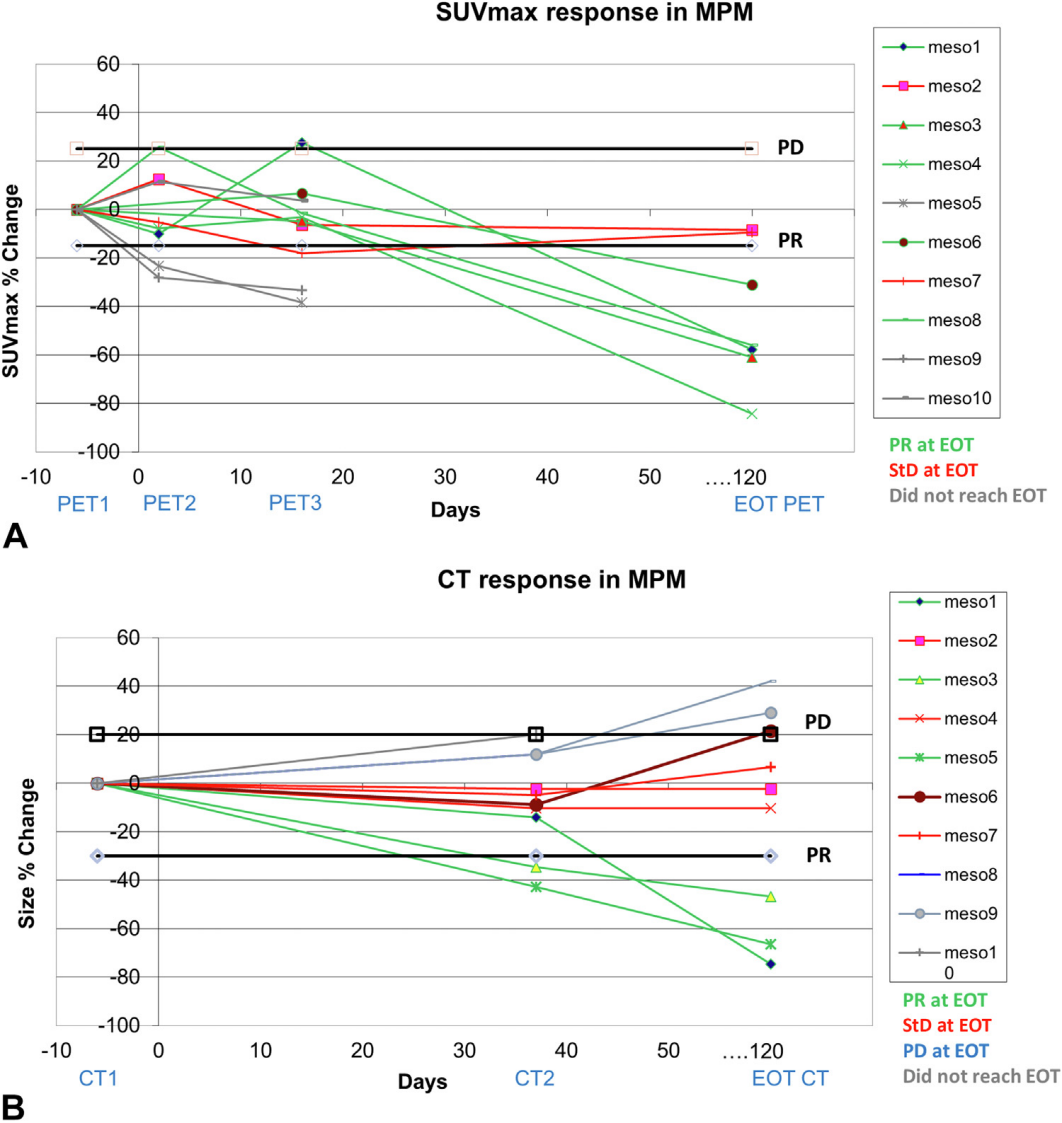
MPM response on (*A*) FLT PET-CT and (*B*) CT. Most MPM cases (71%) had PR at EOT and the remainder (29%) had StD, whereas there is a mixed picture on CT with 33% revealing PR, StD, and PD, respectively. CT, computed tomography; EOT, end of treatment; FLT, 3'-deoxy-3'-fluorothymidine; MPM, malignant pleural mesothelioma; PD, progressive disease; PET, positron emission tomography; PR, partial response; StD, stable disease; SUVmax, maximum standardized uptake value.

**Figure 3:**
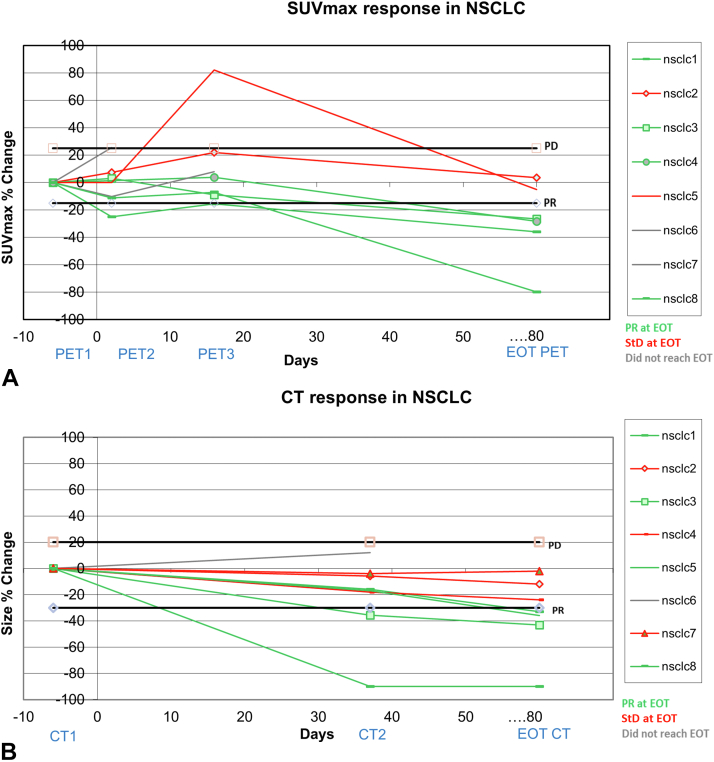
NSCLC response on (*A*) FLT PET-CT and (*B*) CT. Most NSCLC cases (67%) had PR at EOT and the remainder (33%) had StD, whereas CT had PR in 57% and StD in 43%. CT, computed tomography; EOT, end of treatment; FLT, 3'-deoxy-3'-fluorothymidine; PET, positron emission tomography; PR, partial response; StD, stable disease; SUVmax, maximum standardized uptake value.

**Figure 4:**
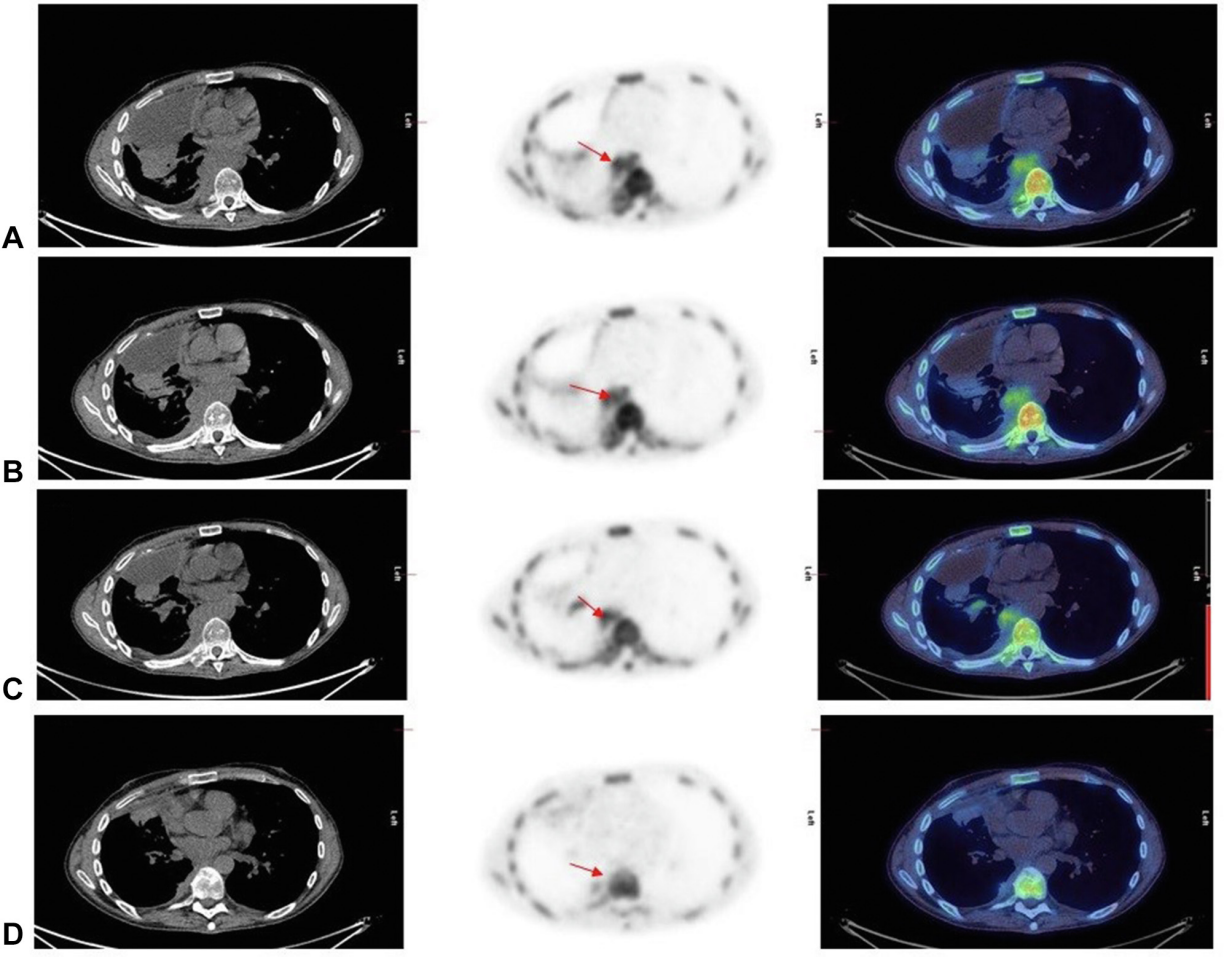
FLT PET-CT in MPM (case 4). (*A*) Baseline SUVmax equals 6.4 (red arrow). (*B*) Post ADI-PEG20 at 24 hours, the SUVmax decreased to 5.9 (8% reduction hence StD). (*C*) Postcycle 1 of combined therapy, the SUVmax increased slightly to 6.2 (maintained StD); however, at (*D*) end of treatment, the SUVmax decreased significantly to 1.0 (84% reduction from baseline hence PR). CT also had response, but StD at EOT and earlier time points. ADI-PEG20, pegargiminase; CT, computed tomography; EOT, end of treatment; FLT, 3'-deoxy-3'-fluorothymidine; MPM, malignant pleural mesothelioma; PET, positron emission tomography; PR, partial response; StD, stable disease; SUVmax, maximum standardized uptake value.

**Figure 5:**
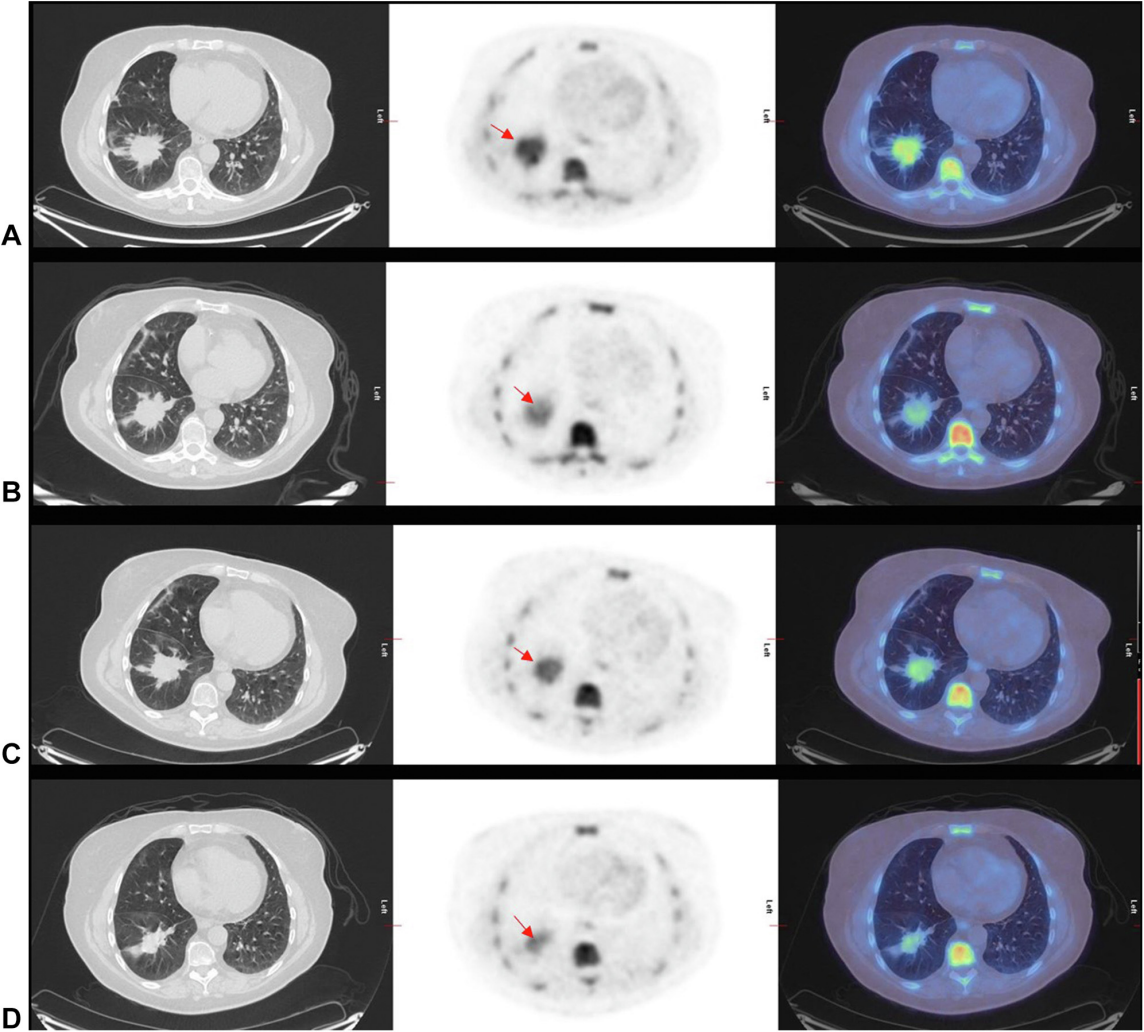
FLT PET-CT in NSCLC (case 11). (*A*) Baseline where primary lesion has SUVmax equals to 6.4 (red arrow). (*B*) Post–ADI-PEG20 at 24 hours, the SUVmax reduced to 4.8 (25% reduction hence PR). (*C*) Postcycle 1 of combined therapy, the SUVmax increased slightly to 5.4; however, at (*D*) end of treatment, the SUVmax decreased further to 4.1 (35% reduction from baseline) and hence PR was maintained. CT similarly had PR at end of treatment with a 33% reduction in size and StD at earlier time points. ADI-PEG20, pegargiminase; CT, computed tomography; EOT, end of treatment; FLT, 3'-deoxy-3'-fluorothymidine; MPM, malignant pleural mesothelioma; PET, positron emission tomography; PR, partial response; StD, stable disease; SUVmax, maximum standardized uptake value.

Table 1 summarizes the response to ADIPemCis treatment by PET as a calculated percentage change in SUVmax from PET1 to time points PET2, PET3, and PET4 and with the corresponding definition of PR, StD, and PD. The table also includes the corresponding change in RECIST size on CE CT (from baseline to EOT) and the OS and PFS in months. It includes data from all 18 participants, although not all participants completed all planned imaging at all time points (two participants did not have EOT CT, four had no PET4, and three had no PET2). These results are also illustrated graphically for MPM (Fig. 2*A* and *B*) and NSCLC (Fig. 3*A* and *B*), with specific patient examples of MPM and NSCLC imaging at various time points illustrated in Figures 4*A–D* and 5*A–D*, respectively.

**Table 1:** Change in SUVmax From Baseline on PET-CT at Time Points PET2 (24 h), PET3 (Post-One Cycle Combined Therapy), and PET4 (EOT); Change in RECIST Size at EOT From Baseline on CT; OS and PFS

Patient	Disease	ASS1% Loss	ΔPET2-PET1	ΔPET3-PET1	ΔPET4-PET1	ΔCT4-CT1	OS/mo	PFS/mo
Case 1	MPM biphasic	80	−10% (StD)	28% (PD)	−58% (PR)	−75% (PR)	18.1	7.6
Case 2	MPM biphasic	70	13% (StD)	−6% (StD)	−8% (StD)	−2% (StD)	9.5	6
Case 3	MPM biphasic	51	No scan	−5% (StD)	−61% (PR)	−47% (PR)	23.1	6.6
Case 4	MPM sarcomatoid	98	−8% (StD)	−3% (StD)	−84% (PR)	−10% (StD)	12.2	2.7
Case 5	MPM biphasic	70	−23% (PR)	−38% (PR)	No scan	−66% (PR)	5.8	2.5
Case 6	MPM biphasic	80	No scan	7% (StD)	−31% (PR)	22% (PD)	12.1	4.3
Case 7	MPM epitheliod	70	−5% (StD)	−18% (PR)	−10% (StD)	7% (StD)	21.3	7.6
Case 8	MPM sarcomatoid	80	26% (PD)	−2% (StD)	−56% (PR)	42% (PD)	10.7	4
Case 9	MPM sarcomatoid	90	−28% (PR)	−33% (PR)	No scan	29% (PD)	3.8	2.7
Case 10	MPM biphasic	100	11% (StD)	4% (StD)	No scan	No scan	2.8	1.2
Case 11	NSCLC	55	−25% (PR)	−16% (PR)	−35% (PR)	−33% (PR)	18.8	7.5
Case 12	NSCLC	80	7% (StD)	22% (StD)	4% (StD)	-12% (StD)	7.4	4.5
Case 13	NSCLC	70	3% (StD)	−9% (StD)	−26% (PR)	−43% (PR)	7.5	3.9
Case 14	NSCLC	100	NO SCAN	4% (StD)	−28% (PR)	−24% (StD)	17.5	11.1
Case 15	NSCLC	80	0% (StD)	82% (PD)	−5% (StD)	−36% (PR)	10.6	6.3
Case 16	NSCLC	98	26% (PD)	No scan	No scan	No scan	2.3	1.8
Case 17	NSCLC	70	−10% (StD)	8% (StD)	No scan	−2% (StD)	6.3	3.3
Case 18	NSCLC	100	−11% (StD)	−7% (StD)	−80% (PR)	−90% (PR)	5.9	4.8

ASS1, argininosuccinate synthetase 1; CT, computed tomography; EOT, end of treatment; MPM, malignant pleural mesothelioma; OS, overall survival; PD, progressive disease; PET, positron emission tomography; PFS, progression-free survival; PR, partial response; RECIST, Response Evaluation Criteria in Solid Tumors; StD, stable disease; SUVmax, maximum standardized uptake value.

Looking at all cases of MPM and NSCLC, there was a mean decrease of 37% plus or minus 16% in SUVmax from PET1 to PET4 (SUV data available in 13 participants of a possible total of 18). The corresponding decrease in RECIST size (mRECIST for MPM and RECIST 1.1 for NSCLC) from CE CT1 to CT4 was 22% plus or minus 14% (in 16 participants). Hence, FLT PET-CT proliferation imaging revealed a large percentage change in parameters of treatment response to ADIPemCis therapy at EOT, in keeping with a PR. This change was greater than the decrease in size found on CE CT at EOT, which in turn revealed StD (as it did not reach the 30% required for PR). At earlier time point of PET scans, there was a mean decrease of 2% in SUVmax from PET1 to PET2 and an increase of 3% in SUVmax from PET1 to PET3. These changes on early PETs were small and within test-retest repeatability metrics. Only PET4 revealed an SUVmax change significantly different from baseline (*p* = 0.002)

Cohen’s kappa test revealed some agreement of k equals to 0.38, with a marginal significance (*p* = 0.043) between PET2 and CT4 across all groups. No statistically significant agreement was observed with PET3 or PET4 with CT. Across all groups, Wilcoxon signed rank tests revealed no statistically significant differences in percentage response between the 3 PET time points and CT4.

### Concordance of Treatment Response on PET-CT and CE CT in Terms of PR, StD, and PD

Looking at treatment response on the EOT CE CT scan (CT4), 7 of 18 participants had PR, 6 of 18 had StD, 3 of 18 had PD, and 2 of 18 had no scan. The response at PET2 (ΔPET2−ΔPET1) was StD in 10 of 18, PR in 3 of 18, PD in 2 of 18, and 3 of 18 did not have a PET2 scan. A total of 13 participants had complete CT4 and PET2 imaging. Response at PET2 agreed with CT4 response in 62% (8 of 13) of the cases overall: 71% (five of seven) in MPM and 50% (three of six) in NSCLC.

If we use a higher cutoff in FLT metrics and define PR as 25% decrease in SUVmax (instead of 15%), only one result in one participant is affected, namely case 5, where response changed from PR to StD at PET2. Even using this higher cutoff, PET2 agreed with CT4 response in 53% (7 of 13) overall and 57% (four of seven) in MPM cases.

### Response Rate on PET-CT and CE CT in Terms of PR, StD, and PD

On the PET-CT data, a PR was found in 20% (3 of 15) of participants at PET2 and 69% (9 of 13) at PET4. On CT, this was lower, namely 44% (7 of 16).

In MPM, a PR was found in 25% (two of eight) at PET2 and increased to 30% (three of 10) at PET3 and to 71% (five of seven) at PET4. PR on CT4 was lower at 33% (three of nine; Figs. 2 and 4). In NSCLC, a PR was found in 14% (one of seven) at PET2 and PET3, but it increased to 67% (four of six) at PET4. PR on CT4 was lower at 57% (four of seven; Figs. 3 and 5).

### Survival Data Analysis

There was no significant correlation of OS and PFS with quantitative percentage response in the whole group or within groups. Cox regression also found no significant relationship between response and either OS or PFS.

## Discussion

In this imaging TRAP substudy, we have assessed tumor proliferation using FLT-PET as a biomarker of treatment response in participants with MPM and NSCLC to arginine-lowering therapy with ADI-PEG 20 combined with pemetrexed and cisplatin; however, we were also able to assess ADI-PEG 20 as a single agent on PET2. We found significant suppression of FLT uptake at the end of ADIPemCis treatment, validating FLT PET-CT as a biomarker in the context of arginine deprivation, while also describing the first use of FLT-PET in MPM. Less robustly—but nonetheless in line with earlier preclinical modeling—we identified reduced FLT uptake by 24 hours of ADI-PEG 20 monotherapy.

Novel mechanistic insights into arginine deiminase pharmacology suggest that FDG lacks specificity for evaluating clinical response in melanoma.^12^ False-positive FDG uptake also occurs with inflammation, owing to activated macrophages, which reveal markedly increased glycolysis.^19^ Thus, participants with MPM, who have undergone talc pleurodesis, may have persistent FDG uptake, making interpretation of tumor from inflammation difficult.^20^ Ceresoli et al.,^21^ in a study of 20 participants with MPM treated with pemetrexed and platinum, found that a decrease in metabolic response determined by SUVmax correlated significantly with time to progression and a trend toward longer survival, whereas response evaluation by CT was not predictive. Veit-Haibach et al.,^22^ however, found that SUVmax was not predictive of survival.

Preclinically, ADI-PEG 20 suppresses both the salvage and de novo synthesis thymidine pathways in ASS1-deficient epithelial tumor cell lines.^7^ Moreover, xenograft studies in epithelial cancer cell lines, but not melanoma cell lines, confirmed that ADI-PEG 20 therapy lowers FLT tumoral uptake thereby providing a rationale for measuring tumor proliferation with FLT PET-CT imaging in clinical participants.^7^^,^^23^ We revealed that a scan as early as 24 hours after ADI-PEG 20 therapy was able to predict EOT RECIST response to ADIPemCis in 62% of cases. A kappa test revealed “fair” agreement of k equals to 0.38, with a marginal significance (*p* = 0.043) between PET2 and CT4. Nevertheless, there was no statistically significant evidence to support that the change in proliferation preceded the change in size (as measured by RECIST). Carlin et al.,^24^ looking at FLT response at day 14 after neoadjuvant chemotherapy in NSCLC, revealed that SUVmax decreased in two of three responders (defined as >30% reduction in unidimensional measurement on CT) and five of six nonresponders.

Frings et al.^25^ assessed FLT PET-CT in pemetrexed-only therapy in NSCLC in 11 participants: two participants had increased FLT uptake (35% and 31%) at 4 hours and two had decreased uptake of 31% (in both) and no change in the remainder. In contrast, we revealed a consistent change at the 24 hours time point, namely decreased or stable FLT uptake at PET2. More recently, FLT PET-CT assessment to novel targeted therapy with a c-MET inhibitor and MDM2 inhibitor in lung cancer revealed an early response at two selected time points, namely 9 days in one participant and at 4 weeks in two other participants.^26^

Antifolates, such as 5-flurouracil, have been found by Perumal et al.^27^ to increase FLT uptake as part of the exogenous (salvage) “flare” response to thymidylate synthase inhibition of the endogenous thymidine pathway, which would be expected also for pemetrexed, a known thymidylate synthase inhibitor.^25^^,^^27^ Nevertheless, xenograft studies have revealed that ADI-PEG 20 suppresses both the thymidine de novo synthesis and salvage pathways, and this effect is maintained in combination with pemetrexed.^7^ Notably, in oncogenic-driven NSCLC, more robust effects compared with arginine deprivation and chemotherapy have been reported during the first few weeks of therapy. For example, a study measuring change in FLT SUVmax between baseline and after 7 days of gefitinib therapy in participants with *EGFR*-mutant lung adenocarcinoma found that responders (as defined on CT evaluation at 6 wk) had a significantly different change in SUVmax than nonresponders (−36% ± 15% versus 10% ± 20%, respectively, *p* < 0.001).^28^

We found that both FLT PET-CT and CT revealed a response to ADIPemCis at the EOT time point. There was a greater change from PET1 to PET4 (mean decrease of 37% ± 16% in SUVmax, n = 13) than on the corresponding CE CTs (22% ± 14% decrease in (m)RECIST length, namely mRECIST in MPM and RECIST 1.1 in NSCLC, n = 16). There were three cases of MPM having PR at CT4, with PR found on the corresponding FLT PET-CT in two cases (no PET scan data in the third); hence, there was an agreement between the mRECIST and SUV measurements. At PET4, there were no cases of PD found on FLT PET-CT and no cases of PD on CT in the NSCLC group alone; whereas 33% (three of nine) of MPM participants did have PD on their final CE CT scan; 19% (3 of 16) when both NSCLC and MPM participants are included. The reason for the differential response in MPM cases on the two modalities is unclear. In the dose-expansion TRAP study, we observed that macrophages increased sevenfold in rebiopsied patients at progression. Macrophages are nonproliferative but expand the tumor volume,^14^ potentially increasing mRECIST size with no change in FLT uptake and proliferation.

Furthermore, p53 mutations, characteristic of nonepithelioid MPM, were also 40% higher than that expected in the nonsquamous NSCLC expansion cohort and may also affect FLT PET-CT–based imaging.^29^ In addition, steroid (dexamethasone) prophylaxis was omitted in the TRAP study before PET2 until after the scan had been completed; recent preclinical and clinical work has confirmed significant reductions in FLT tracer uptake with 24 hours of dexamethasone treatment in NSCLC.^15^^,^^30^ Hence, although FLT-PET imaging lacks robustness for early time point imaging, this study provides evidence for its use in the evaluation of the arginine-ASS1-ADI pathway in thoracic cancers, especially in MPM. Indeed, in view of the recognized constraints of mRECIST, further testing of FLT-PET as an imaging tool would be indicated for MPM treatment response assessment.

In the MPM expansion cohort of the TRAP study (n = 31, including the 10 participants enrolled in the PET substudy), we found a disease control rate of 94% and a PR rate of 36% at 18 weeks. The median PFS and OS were 5.6 and 10.1 months, respectively.^14^ Similarly, there was a high disease control rate of 86% in the nonsquamous NSCLC expansion cohort of the TRAP study (n = 21), including the eight participants enrolled in the PET substudy), with a PR rate of 48% at 12 weeks. Here, the median PFS and OS were 4.2 (95% confidence interval: 2.9–4.8) and 7.2 (95% confidence interval: 5.1–18.4) months, respectively, and consistent with the poor prognosis of ASS1-deficient cancers.^15^ Some cancer which initially have a PR can progress quickly. For example, case 5 who had a decrease in RECIST length of 66% at EOT progressed at only 2.5 months. This is considered to be on account of the p53 mutation, causing early resistance to arginine therapy. FLT-PET imaging provides additional validation of arginine auxotrophic thoracic cancers, especially MPM, in which there are several resistance mechanisms, including ASS1 re-expression (i.e., recycling of citrulline to arginine), autophagy, and metabolic support by macrophages and other stromal cells, which affect subsequent disease progression.^31^

Early phase combination trials of ADI-PEG 20 with chemotherapy are reporting increased efficacy, owing to synergistic and additive mechanisms of cytotoxicity in various cancer types, accompanied by more sustained arginine depletion with the slower emergence of anti–ADI-PEG 20 antibodies.^13^^,^^32^, ^33^, ^34^ In thoracic cancers, this multimodality strategy has progressed to a randomized phase 3 study of ADI-PEG 20 (or placebo) PemCis focusing on chemorefractory (nonepithelioid) MPM which is expected to report final results in 2022 (ATOMIC-meso, ClinicalTrials.gov identifier: NCT02709512). The current substudy therefore provides evidence supporting arginine deprivation with pegargiminase in targeting thymidine uptake as a treatment for ASS1-deficient thoracic tumors.

### Limitations

We do not have FLT test-retest data in this study, but previous studies have revealed this to be good (test-retest *r* ≥ 0.97 on serial baseline scans in a study on breast cancer).^35^ Some test-retest data looking specifically at FLT imaging in solid tumors suggest that a higher percentage change (≥25%) in FLT SUV metrics may be needed to represent a true change in tumor uptake.^36^ Quantitative FLT measurements are reproducible in NSCLC, and when monitoring response in individual patients, changes of more than 20% to 25% in SUV(max) are likely to represent treatment effects.^37^ There are no data looking specifically at FLT measurements in MPM. We found that only one result changed if we used a 25% rather than 15% cutoff to define PR and although EORTC criteria have not been validated for FLT imaging, percentage change from baseline has been described in multiple studies as a measure of response; hence, we used EORTC-based criteria in this study. No other validated measure was available. Positron Emission Tomography (PET) Response Criteria in Solid Tumors (PERCIST 1.0) was not suitable owing to the high background hepatic uptake and is also not validated except for FDG in solid tumors.^18^

A further limitation of our study is that we are relying on RECIST data for CT measurements, as there was no better surrogate marker of response available. In addition, the number of participants is also too small to reliably compare to PFS and OS and may have limited the ability to determine differences statistically. These participants are typically unwell symptomatically owing to the significant burden of disease, and thus recruitment is challenging. This was a longitudinal study, and a number of participants were unable to complete the full imaging protocol owing to morbidity.

A more general limitation of using FLT in imaging treatment response is that it evaluates the exogenous thymidine pathway only; there is a potential “flare” from increased dependence on exogenous thymidine after antifolate therapy although this was not found in the preclinical studies^7^; and there is a potential increase in unconjugated FLT in plasma (as some chemotherapy agents deplete glucouronidate and hence less FLT is conjugated with a resultant increase in FLT plasma fraction).

Although we have found evidence to support FLT-PET imaging in the evaluation of the arginine-ASS1-ADI pathway in thoracic cancers (especially MPM), the small sample size, high dropout, wide confidence intervals, and barely significant *p* values suggest that further evaluation is warranted.

### Conclusions

Our study reveals that reduction of FLT uptake by 24 hours of ADI-PEG 20 monotherapy in line with preclinical modeling and early FLT imaging agrees with the EOT CT results in nearly two-thirds of participants, with k equal to 0.38 (*p* = 0.043). There is robust suppression of FLT uptake at the end of ADIPemCis treatment with an overall mean decrease of 37% in SUVmax. We have described the first-in-human use of FLT PET-CT in the assessment of treatment response in MPM, revealing a PR rate of 25% (two of eight) at 24 hours and 71% (five of seven) at EOT, which is higher than the PR rate on the EOT CT, at 33% (three of nine). Although on the basis of a limited number of participants, the robust EOT scan is a promising alternative to mRECIST for future response assessment in MPM.

## CRediT Authorship Contribution Statement

**Teresa A. Szyszko:** Writing—original draft, Investigation, Formal analysis, Data curation.

**John Bomalaski:** Conceptualization, Project administration.

**Gary J. R. Cook:** Supervision, Writing—review and editing, Visualization.

**Joel T. Dunn:** Statistics, Methodology, Software, Formal analysis.

**Steve Ellis**: Investigation.

**Vicky Goh:** Supervision, Writing—review and editing.

**Melissa M. Phillips:** Methodology.

**Lucy Pike:** Software, Resources.

**Michael T. Sheaff:** Histology, Investigation.

**Peter W. Szlosarek:** Supervision, Writing—review and editing, Conceptualization.
