# Supplementary material for: Role of 3'-Deoxy-3'-[18F] Fluorothymidine Positron Emission Tomography-Computed Tomography as a Predictive Biomarker in Argininosuccinate Synthetase 1-Deficient Thoracic Cancers Treated With Pegargiminase
Source: JTO Clin Res Rep. 2022 Jul 20;3(9):100382. doi: 10.1016/j.jtocrr.2022.100382 (PMC9445378; doi:10.1016/j.jtocrr.2022.100382)
Supplement: Supplementary Data [file mmc1.docx]

# **Supplementary Data**

Table of Demographic data

| **MPM Characteristic** | **Number** | **NSCLC Characteristic** | **Number** |
| --- | --- | --- | --- |
| **Age/years** |  | **Age/years** |  |
| mean (range) | 69 (58-82) | mean (range) | 58 (39-65 |
| **Gender** |  | **Gender** |  |
| male | 9 | female | 4 |
| female | 1 | male | 4 |
| **Race** |  | **Race** |  |
| Caucasian | 10 | Caucasian | 6 |
| Afro-Caribbean | 0 | Afro-Caribbean | 2 |
| **Stage ^*^** | IB-IV | **Stage ^*^** | IV |
| **Histology** |  | **Histology** |  |
| biphasic | 5 | adenocarcinoma | 7 |
| sarcomatoid | 4 | pleomorphic (giant cell) | 1 |
| epithelioid | 1 |  |  |

*Eighth AJCC TNM classification
